# Pro-inflammatory TNF-α and IFN-γ Promote Tumor Growth and Metastasis via Induction of MACC1

**DOI:** 10.3389/fimmu.2020.00980

**Published:** 2020-05-27

**Authors:** Dennis Kobelt, Chenyu Zhang, Isabelle Ailish Clayton-Lucey, Rainer Glauben, Cynthia Voss, Britta Siegmund, Ulrike Stein

**Affiliations:** ^1^Experimental and Clinical Research Center, Charité Universitätsmedizin Berlin and Max-Delbrück-Center for Molecular Medicine in the Helmholtz Association, Berlin, Germany; ^2^German Cancer Consortium (DKTK), Partner Site Berlin, and German Cancer Research Center (DKFZ), Heidelberg, Germany; ^3^Charité – Universitäsmedizin Berlin, Corporate Member of Freie Universität Berlin, Humboldt-Universität zu Berlin, and Berlin Institute of Health, Department of Gastroenterology, Infectious Diseases and Rheumatology, Campus Benjamin Franklin, Berlin, Germany

**Keywords:** MACC1, metastasis, TNF-α, pro-inflammatory cytokines, colorectal cancer

## Abstract

Colorectal cancer (CRC) is one of the most common malignancies worldwide. Early stage CRC patients have a good prognosis. If distant metastasis occurs, the 5-year survival drops below 10%. Despite treatment success over the last decades, treatment options for metastatic disease are still limited. Therefore, novel targets are needed to foster therapy of advanced stage CRC patients and hinder progression of early stage patients into metastasis. A novel target is the crucial oncogene Metastasis-Associated in Colon Cancer 1 (MACC1) involved in molecular pathogenesis of CRC metastasis. MACC1 induces cell proliferation and motility, supports cellular survival and rewires metabolism resulting in increased metastasis *in vivo*. MACC1 is a prognostic biomarker not only for CRC but for more than 20 solid cancer entities. Inflammation plays a pivotal role in tumorigenesis, tumor progression and metastasis. For CRC, inflammatory bowel disease and ulcerative colitis are important inflammation associated risk factors. Certain cytokines, such as TNF-α and IFN-γ, are key factors in determining the contribution of the inflammatory process to CRC. Knowledge of the connection between inflammation and MACC1 driven tumors remains unclear. Gene expression analysis of CRC cells after cytokine stimulation was analyzed by qRT-PCR and Western blot. Cellular motility was assessed by Boyden chamber assays. MACC1 promoter activity after stimulation with pro-inflammatory cytokines was measured using promoter-luciferase constructs. To investigate signal transduction from receptor to effector molecules, blocking experiments using neutralizing antibodies and knockdown experiments were performed. Following TNF-α stimulation, MACC1 and c-Jun expression were significantly increased at the mRNA and protein level. Knockdown of c-Jun reduced MACC1 inducibility following TNF-α stimulation. TNF-α promoted MACC1-induced cell migration that was reverted following MACC1 knockdown. Moreover, MACC1 and c-Jun expression were downregulated by blocking TNFR1, but not TNFR2. Knock down of the NF-κB subunit, p65, reduced basal MACC1 and c-Jun mRNA expression levels. Adalimumab, a clinically approved monoclonal anti-TNF-α antibody, hindered MACC1 induction. The present study highlights that TNF-α regulates the induction of MACC1 via the NF-κB subunit p65 and the transcription factor c-Jun in CRC cells. This finding unravels a novel signaling pathway upstream of MACC1 and provides a potential therapeutic target for the treatment of CRC patients with an associated inflammation.

## Introduction

Inflammation is a defense mechanism of the immune system of higher multicellular organisms (1). It is triggered by stimuli including pathogens, injuries, chemicals or radiation (2). The protective responses are essentially connected to the healing process after the trigger is removed (3). Inflammation is mediated and controlled by different cell types and secreted proteins including pro-inflammatory cytokines (4). The major pro-inflammatory cytokines in different diseases are TNF-α and IFN-γ (5–7). Both belong to the group of immune modulating molecules that act through specific cell-surface receptors and participate in autocrine, paracrine and endocrine signaling (8–11). They modulate the innate and adaptive immune system (4, 12). More importantly, they are also associated with chronic inflammation and represent crucial factors in tumor development (13–15). Chronic inflammation is known as causal risk factor for tumor development, but the intimate connection of inflammation and tumor development at the molecular level is still only partly understood.

Colorectal cancer (CRC) is a major cause of morbidity and mortality worldwide and especially in developed contries (16, 17). It contributes to more than 8% of all cancer incidences that affect both men and women, making it the third most common cancer globally (18). There are numerous risk factors for CRC like diet, “Western lifestyle,” excessive alcohol and tobacco intake and, environmental exposure (19–22). Diseases like ulcerative colitis and Crohn's disease connect the formation of sporadic CRC and chronic inflammatory conditions (23–25). Sporadic CRC accounts for the majority of all CRC cases. A smaller fraction of about 10–15% of all CRC cases is based on hereditary risk factors like in familial adenomatous polyposis (FAP) and hereditary nonpolyposis colorectal cancer (HNPCC) (26, 27). There is growing evidence that inflammation is not only connected to sporadic cases of CRC but that reduced inflammatory responses can equally reduce or delay the formation of hereditary CRCs (27, 28). Ulcerative colitis is responsible for 1% of all CRC cases due to chronic inflammation affecting the mucosa of the colon and rectum, and Crohn's disease and here in particular Crohn's colitis has also been shown to slightly increase the risk (16). Inflammation is causing differential gene expression for a broad spectrum of genes. Therefore, it is needed to understand, which of these genes are the most important drivers of CRC and might serve as biomarkers and as therapeutic targets in patient tailored treatments.

One such driver of tumor progression is the gene Metastasis-Associated in Colon Cancer 1 (MACC1). The importance of MACC1 has been first demonstrated in CRC as prognostic marker of metastasis formation and metastasis-free survival (29). Both MACC1 mRNA and protein are highly expressed in CRC tissues with metachronous metastases compared to tumors without metastases and to normal tissue. The expression of MACC1 is increased during the transition from adenomas to carcinomas (30, 31). This suggests that MACC1 represents an independent early prognostic marker for CRC metastasis (32, 33). Besides CRC, MACC1 is meanwhile also a prognostic marker for more than 20 solid tumor entities (34).

MACC1 is a causal driver of tumor progression and metastasis. The reason for the increased MACC1 gene expression is largely unknown. Here we analyzed the connection of inflammation and MACC1 expression in the context of pro-inflammatory cytokines.

## Materials and Methods

### Immunhistochemical Staining of MACC1 in Patient Paraffin Tissue Sections

Written informed consent was obtained from all patients. All experiments were approved by the institutional review board of the Charité–Universitätsmedizin Berlin and conducted accordingly. The authors complied with all relevant ethical regulations for research involving human participants. MACC1 protein expression was assessed in 14 tissue samples (five male, nine female patients, median age 55.5 years) of ulcerative colitis and Crohn's disease patients.

For paraffin removal and antigen retrieval tissues were treated with Xylol, 2:1 vol/vol aceton/Tris and finally boiled in 10 mM citrate buffer pH 6.3. Specimens were blocked for 30 min at room temperature with horse serum and incubated with primary MACC1 antibody for 2 h (HPA020103, Sigma Aldrich, Munich, Germany). After washing, the slides were incubated with a biotinylated secondary anti rabbit antibody (30 min, room temperature) and streptavidin-peroxidase (VECTASTAIN Elite ABC HRP Kit, PK-6101 Vector Laboratories, Burlingame, CA, USA) for another 30 min at room temperature. Finally, staining was visualized with 3,3′-diaminobenzidin (DAB Peroxidase (HRP) Substrate Kit, SK-4100, Vector Laboratories) and nuclei were stained with haemalaun. The tissues were photographed using a magnification of 100 x for the overviews and 400 x for the insets.

### Cell Culture

HCT116 (LGC Standards, Wesel, Germany) human CRC cells were cultured at 37°C, 100% atmospheric humidity and 5% CO_2_ in RPMI (Thermo Fisher Scientific, Waltham, MA, USA) –emented with 10% fetal calf serum (Bio&Sell, Feucht, Germany). Cells were harvested using trypsin/EDTA (Thermo Fisher Scientific) and counted in an automated cell counter (NanoEnTek, Seoul, Korea). Cells were regularly verified as mycoplasma-negative (Lonza, Basel, Switzerland). Authentication of cell lines was performed by short tandem repeat (STR) genotyping (Multiplexion, Heidelberg, Germany). STR genotypes were consistent with published genotypes.

### Cytokine Treatment

Recombinant human TNF-α and IFN-γ (Peprotech, Hamburg, Germany) were stored at −20°C following reconstitution to 0.1 mg/ml in sterile, deionized water. To maintain the stability of the cytokines, small aliquots were created for single use. Briefly, 1 × 10^6^ cells/well were seeded in 6-well plates and allowed to adhere for 24 h. Subsequently, cells were treated with increasing concentrations (1, 10, 100 ng/ml) of cytokines and harvested after 24 and 48 h. Each experiment was performed in triplicate.

### siRNA Transfection

Preestablished siRNAs targeting c-Jun (Thermo Fisher Scientific), p65 (kind gift of Prof. Claus Scheidereit, Max-Delbrück-Center for Molecular Medicine, Berlin, Germany), as well as scrambled siRNA (Thermo Fisher Scientific) serving as a negative control, were used. 3 × 10^5^ HCT116 cells were seeded in 6-well plates and cultured for 24 h. siRNAs were transfected using the RNAiMAX RNAiMAX transfection reagent following manufacturer's recommendations. Cells were harvested after incubation for 24 and 48 h. Experiments were performed in three biological replicates.

### Plasmid Transfection

To analyze MACC1 promoter activity, pGL4.17-based (Promega, Fitchburg, WI, USA) promoter reporter constructs generated earlier were transfected prior to TNF-α treatment into HCT116 cells (35). Prior transfection using TransIT 2020 (Mirus, Madison, WI, USA) following manufacturer's recommendations, 7.5 × 10^4^ cells were seeded into 24-well plates and allowed to adhere for 24 h. To normalize for transfection efficiency, the pGL4.74 (Promega) encoding for renilla luciferase plasmid was transfected in parallel. Following addition of the transfection complex the cells were grown for 24 h before TNF-α treatment started.

### Dual Luciferase Reporter Gene Assay

The activities of the firefly and renilla luciferases were measured using the Dual-Luciferase reporter assay system (Promega). Cells transiently expressing the luciferase constructs were lysed in passive lysis buffer with gentle shaking for 15 min at room temperature. Equal amounts of lysate and luciferase substrate were added to 96-well luminescence plates (Corning, Corning, NY, USA). The firefly luminescence was quantified first using an Infinite M200 pro 96-well plate reader (Tecan, Männedorf, Switzerland). Following addition of the Stop&Glo reagent, the renilla luciferase luminescence was assessed. Firefly luciferase activities were normalized to renilla luciferase readings.

### Cell Migration

For the evaluation of cell migration, the Boyden chamber assay was used. Membrane inserts (Sigma) with a pore size of 8 μm were used in 24-well plates. Cells were serum-starved overnight. The following day, 600 μl medium containing 10% FCS, without or with increasing amounts TNF-α (1, 10, 100 ng/ml), were added to each lower chamber. Then, 3 × 10^5^ cells in 300 μl medium containing 1% FCS, without or with increasing amounts of TNF-α (1, 10, 100 ng/ml), were seeded into each transwell upper chamber. Cells were incubated for 24 h to allow migration. The cells that had migrated to the lower side of the membrane were harvested with trypsin/EDTA and pooled with the cells in the lower chamber prior to centrifugation (200x g, 5 min at room temperature). To analyze relative cell numbers the cell titer-glo reagent (Promega) was used. After incubation for 10 min in the dark, luminescence intensity was measured with an Infinite M200 pro 96-well plate reader. Each migration assay was performed three times in triplicate.

### TNF-α and Adalimumab or TNFR Antibody Treatment

Adalimumab (HUMIRA®, Il, USA, 100 mg/ml) was stored at 4°C. For TNF-α treatment of HCT116 cells, 2 × 10^5^ cells were plated in 6-well plates and allowed to adhere for 24 h. Then, TNF-α was diluted in RPMI media and added to fresh cell media. Sterile water served as control treatment. For co-treatment, TNF-α and Adalimumab were added to fresh RPMI 10% FBS media to achieve a final concentration of 10 ng/ml TNF-α and 1, 10, or 100 μg/ml Adalimumab. The cells were then incubated for 24 h at 37°C with 5% CO_2_ before harvesting for RNA and protein isolation. For experiments blocking TNFR1 or TNFR2, cells were pretreated with the respective antibodies (TNFR1: MAB225-100 R&D; TNFR2: MAB726-100, R&D Systems, MN, USA) 1 h before adding 10 ng/ml TNF-α.

### Total RNA Isolation, cDNA Synthesis and Quantitative Real-Time PCR

The total RNA was isolated using the GeneMatrix Universal RNA Purification Kit (Roboklon, Berlin, Germany), according to the manufacturer's instructions. Briefly, cells were harvested, lysed and applied to the columns. After washing the columns RNA was eluted with 50 μl nuclease-free H_2_O. RNA concentration was quantified using a NanoDrop spectrophotometer (Thermo Fisher Scientific). The samples were stored at −80°C until further use.

For reverse transcription 50 ng total RNA was used. Reverse transcription was performed with 2.5 μM random hexamers in 5 mM MgCl_2_, 1x PCR buffer, 4 mM dNTPs pool, 1 U/μl RNAse inhibitor and 2.5 U/μl MuLV reverse transcriptase (all Thermo Fisher Scientific). The reaction was carried out at 42°C for 45 min, 99°C for 5 min and 5°C for 5 min. cDNA was stored at −20°C until use.

Quantitative PCR was performed using SYBR Green dye chemistry (GoTaq qPCR Master Mix, Promega) in a LightCycler 480 II system (Hoffmann—La Roche, Basel, Switzerland). The data were evaluated by the LightCycler 480 Software release 1.5.0 SP3. All primer sequences are summarized in Table 1.

**Table 1:** Primer used for qRT-PCR.

**Gene**	**Sequence**
MACC1 F	5′− TTCTTTTGATTCCTCCGGTGA−3′
MACC1 R	5′− ACTCTGATGGGCATGTG TG−3′
c-Jun F	5′− CAGGTGGCACAGCTTAAACA−3′
c-Jun R	5′− GTTTGCAACTGCTGCGTTAG−3′
Sp1 F	5′− GCTCTGAACATCCAGCAAAA−3′
Sp1 R	5′− CAGAGTTTGGAACAGCCTGA−3′
p65 F	5′− ACAACCCCTTCCAAGTTCCT−3′
p65 R	5′− ATCTTGAGCTCGGCAGTGTT−3′
GAPDH F	5′− GAAGATGGTGATGGGATTTC−3′
GAPDH R	5′− GAAGGTGAAGGTCGGAGT−3′
G6PDH F	5′− ATCGACCACTACCTGGGCAA−3′
G6PDH R	5′− TTCTGCATCACGTCCCGGA−3′

*F, forward; R, reverse*.

### Protein Extraction and Western Blotting

For total protein extraction, harvested and washed cells were lysed in RIPA buffer supplemented with cOmplete Protease Inhibitor Cocktail (Sigma) for 15 min on ice. Supernatants were collected following centrifugation at 20,000x g for 20 min at 4°C and stored at −80°C until further use.

The protein concentration of the supernatant was determined by a bicinchoninic acid (BCA) protein assay (Thermo Fisher Scientific) according to the manufacturer's instructions. The lysates were diluted in PBS, and quantified relative to a BSA standard curve. The absorbance was measured at 560 nm using the Tecan Infinite M200 pro.

For Western blotting, 20 μg total protein was mixed with 1x NUPAGE sample buffer (Thermo Fisher Scientific), supplemented with 10% DTT, and heated for 10 min at 95°C. Proteins were separated on 10% NuPAGE Bis-Tris gels (Thermo Fisher Scientific) in 500 ml MOPS buffer (Carl Roth, Karlsruhe, Germany) at 150 V for 1 h. The proteins were then transferred to a nitrocellulose membrane using a semi-dry turbo-blot (Bio-Rad, Hercules, CA, USA) electrotransfer apparatus. After blocking the membrane in 5% skimmed milk powder (Carl Roth) in TBST for 1 h at room temperature, the membrane was incubated with primary antibodies at 4°C overnight (rabbit anti-MACC1, HPA020081, Sigma; rabbit anti-c-Jun, 60A8, Cell Signaling; mouse anti-β-actin, A1978, Sigma). Protein bands were visualized with a suitable horseradish peroxidase conjugated secondary antibodies (anti-rabbit IgG-HRP, W4011, Promega; anti-mouse IgG-HRP, 31430, Thermo) and WesternBright ECL (Biozym, Hessisch Oldendorf, Germany) substrate. Light emission was documented using Fuji medical X-Ray films (Kisker Biotech, Schweinfurt, Germany).

### Statistical Analysis

Statistical analysis was performed using GraphPad Prism Version 6 (GraphPad Software, San Diego, CA, USA). Comparisons of controls with multiple experimental groups were carried out using one-way analysis of variance (ANOVA) followed by a Dunnett *post-hoc* test. Statistical significance was defined for *p*-values below 0.05, with ^*^p ≤ 0.05, ^*^p ≤ 0.01 and ^***^p ≤ 0.001 and ^****^p ≤ 0.0001.

## Results

### MACC1 Protein Level Is Increased in Inflamed Patient Tissue

**Figure 1:**
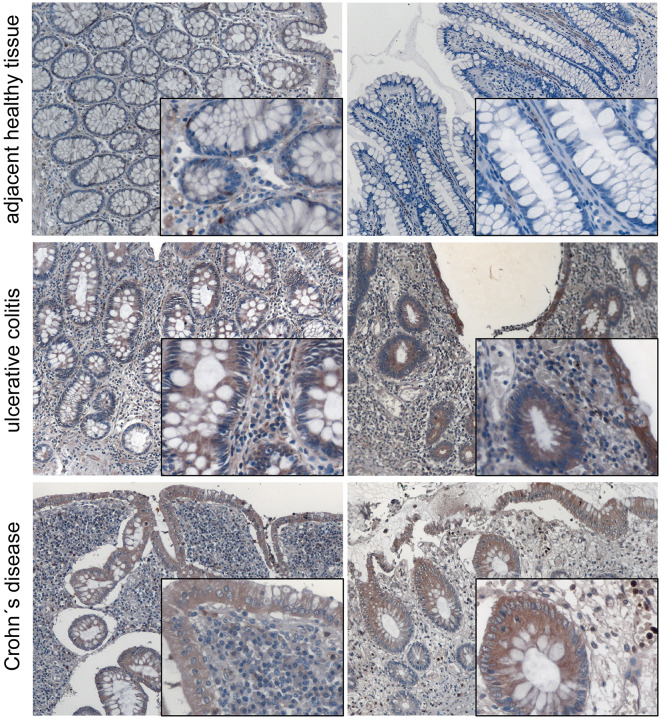
MACC1 protein expression is increased in inflamed tissue. MACC1 protein expression was assessed in 14 tissue samples (five male, nine female patients, median age 55.5 years) of ulcerative colitis and Crohn's disease patients. Besides typical signs of extensive inflammation, areas of actively inflamed tissue show moderate to strong MACC1 staining especially in epithelial tissue compared to adjacent healthy tissue. The tissues were photographed using a magnification of 100 x for the overviews and 400 x for the insets.

We and other groups have shown that MACC1 expression levels are increased especially in tumor tissue of patients with poor outcome (34). For CRC it was shown that MACC1 occurs very early during the transition from adenoma to carcinoma. In order to provide insights of MACC1 gene expression in inflamed tissue before tumor development we stained tissues from ulcerative colitis and Crohn's disease patients for MACC1. A pathologist confirmed active inflammation and evaluated the microphotographs. Specimens of non-inflamed tissue showed weak MACC1 expression only (Figure 1). By contrast, inflamed tissues from ulcerative colitis and Crohn's disease patients revealed moderate to strong MACC1 expression mainly in the cytoplasm of the cells (Figure 1), indicating the association of chronic inflammation and increase in MACC1 expression. Tissues outside of inflamed areas of ulcerative colitis and Crohn's disease patients served as controls.

### TNF-α and IFN-γ Regulate MACC1 mRNA and Protein Expression Levels

**Figure 2:**
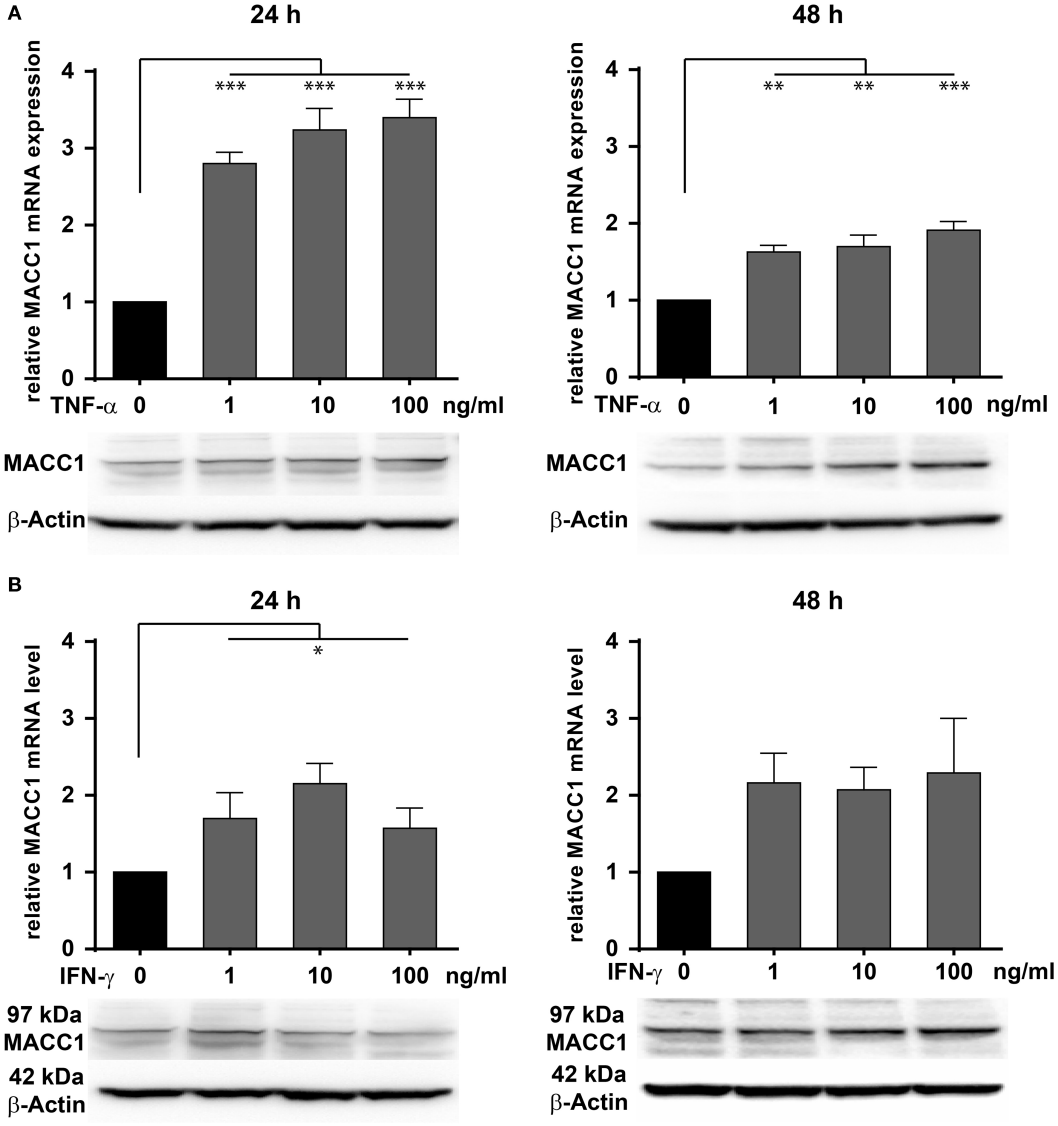
Effects of TNF-α and IFN-γ stimulation on the MACC1 gene expression. HCT116 cells were treated with increasing concentrations of TNF-α (1, 10, 100 ng/ml) **(A)** and IFN-γ (1, 10, 100 ng/ml) **(B)** for 24 h (left side) and 48 h (right side). Cells without cytokine treatment served as controls. MACC1 mRNA expression levels were determined by qRT-PCR and normalized to GAPDH. Evaluation of MACC1 protein expression levels was performed by Western blot, and β-actin served as loading control. Both pro-inflammatory cytokines can upregulate MACC1 gene expression in a dose- and time-dependent manner. This effect was more pronounced for TNF-α. All experiments were performed as three biologically independent experiments. The data are presented as mean ± SEM with the statistical significance levels: *p ≤ 0.05; **p ≤ 0.01; ***p ≤ 0.001.

To evaluate the effect of inflammation on MACC1 in epithelial CRC cells, we assessed the impact of two major pro-inflammatory cytokines, TNF-α and IFN-γ on MACC1 expression. The CRC cell line HCT116 was treated with increasing concentrations of either TNF-α (Figure 2A) or IFN-γ (Figure 2B) for 24 and 48 h, respectively. The mRNA and protein expression levels of MACC1 were determined by qRT-PCR and Western blot.

Compared with the untreated control cells, MACC1 mRNA expression levels were significantly increased by 3-fold upon treatment with 1 ng/ml (*p* < 0.05), 10 ng/ml (*p* < 0.01), and 100 ng/ml (*p* < 0.01) TNF-α (Figure 2A, left panel). Following 48 h of treatment, the increase in mRNA expression levels of MACC1 declined but was still significantly elevated by 1.5- to 2-fold. Consistent with the increase in mRNA expression levels, MACC1 protein expression was also upregulated following 24 and 48 h TNF-α treatment in a dose-dependent manner. This finding was confirmed in three different established cell lines and three different primary cell models (Supplementary Figure 1).

Similarly, HCT116 cells were exposed to increasing concentrations of IFN-γ for 24 and 48 h (Figure 2B). MACC1 mRNA and protein expression levels were determined by qRT-PCR and Western blotting, respectively. For this cytokine, the increase in the MACC1 mRNA levels was still there but not as pronounced as for TN-α treatment. These experiments demonstrate that stimulation with pro-inflammatory cytokines was able to upregulate MACC1 mRNA and protein expression in a dose- and time-dependent manner.

### TNF-α and IFN-γ Induce Cell Migration

**Figure 3:**
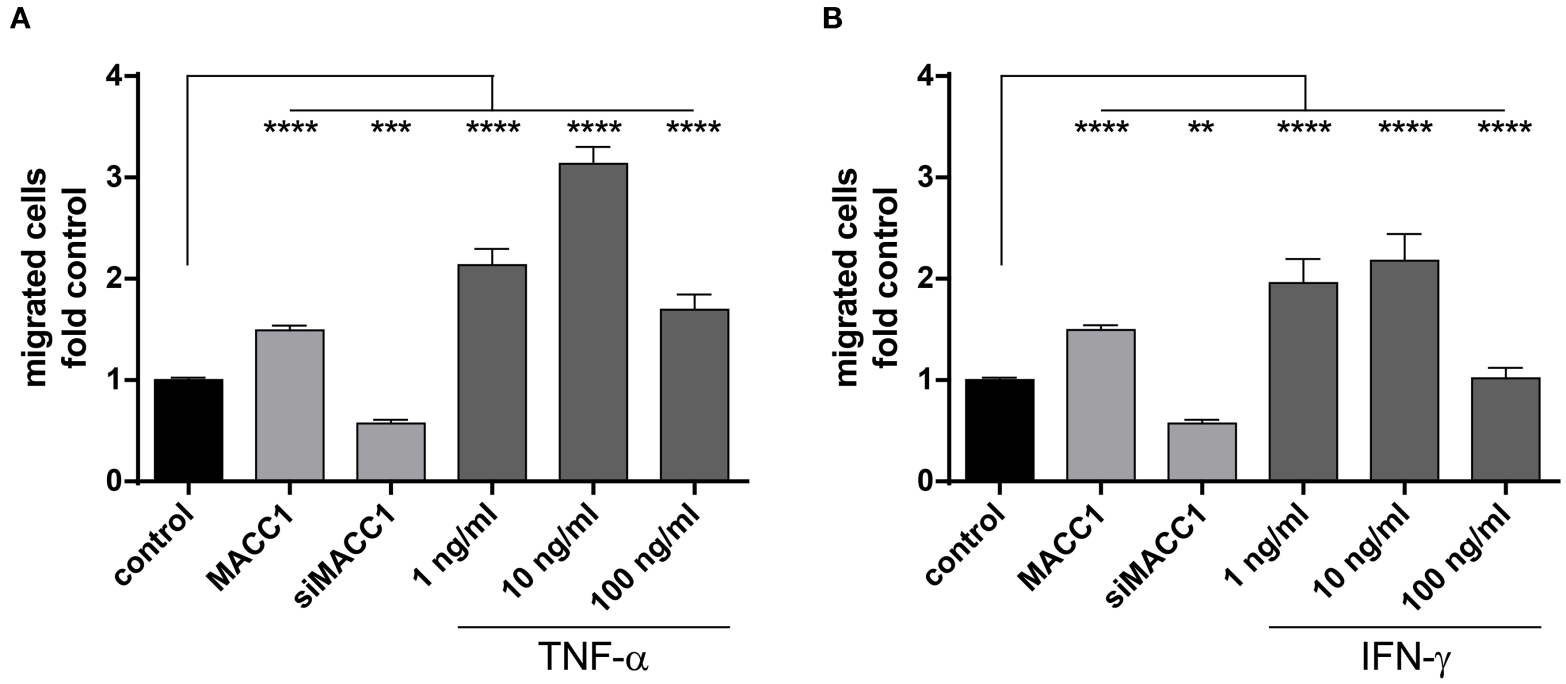
MACC1 induced by pro-inflammatory cytokines increases migration. MACC1-dependent cell migration was confirmed by stable MACC1 overexpression or MACC1 siRNA-mediated MACC1 down-regulation. Cells were treated with either TNF-α **(A)** of IFN-γ **(B)** for 24 h before cell migration was measured. Results are representative of at least four independent experiments. The data are presented as mean ± SEM with the statistical significance levels: **p ≤ 0.01; ***p ≤ 0.001 and ****p ≤ 0.0001.

As shown above, exposure to TNF-α induces MACC1. To explore, if this increased MACC1 expression results in increased migratory potential of cells, we tested HCT116 cells in the Boyden chamber assay. First, we confirmed MACC1-dependent changes in migration by either overexpressing MACC1 by stable transfection or specific downregulation of MACC1 by siRNA. Cell migration was increased with elevated MACC1 expression and decreased if MACC1 was knocked down by siRNA (Figures 3A,B). Treatment with increasing concentrations of TNF-α (1, 10, or 100 ng/ml) was performed for 24 h. TNF-α induced cell migration by more than 2-fold at a concentration of 1 ng/ml (Figure 3A), compared with unstimulated cells. Upon treatment with 10 ng/ml TNF-α, cell migration was even stronger induced by 3-fold in HCT116 cells, compared with control cells. Interestingly, at a concentration of 100 ng/ml TNF-α, cell migration was not as strongly induced as at lower TNF-α concentrations but still elevated above control levels. To confirm this we tested in addition to the Boyden chamber migration assay cellular motility in the wound healing (scratch) assay. TNF-α induced faster wound closure compared to control cells (Supplementary Figure 2A). The data clearly indicates that TNF-α was able to induce cell migration *in vitro* in a dose-dependent manner. To determine the role of the pro-inflammatory cytokine IFN-γ on cell migration, HCT116 cells were treated with increasing concentrations of IFN-γ. This cytokine induced cell migration by 2-fold at concentrations of 1 as well as 10 ng/ml as compared with the unstimulated control cells (Figure 3B). However, the treatment with 100 ng/ml IFN-γ did not result in significant changes of cell migration. The data show that pro-inflammatory cytokines induce cell migration that is paralleled by an increased MACC1 expression. This was most efficient at lower concentrations of TNF-α and the effect of TNF-α was more pronounced than the effect of IFN-γ.

### TNF-α Induces MACC1 via c-Jun

**Figure 4:**
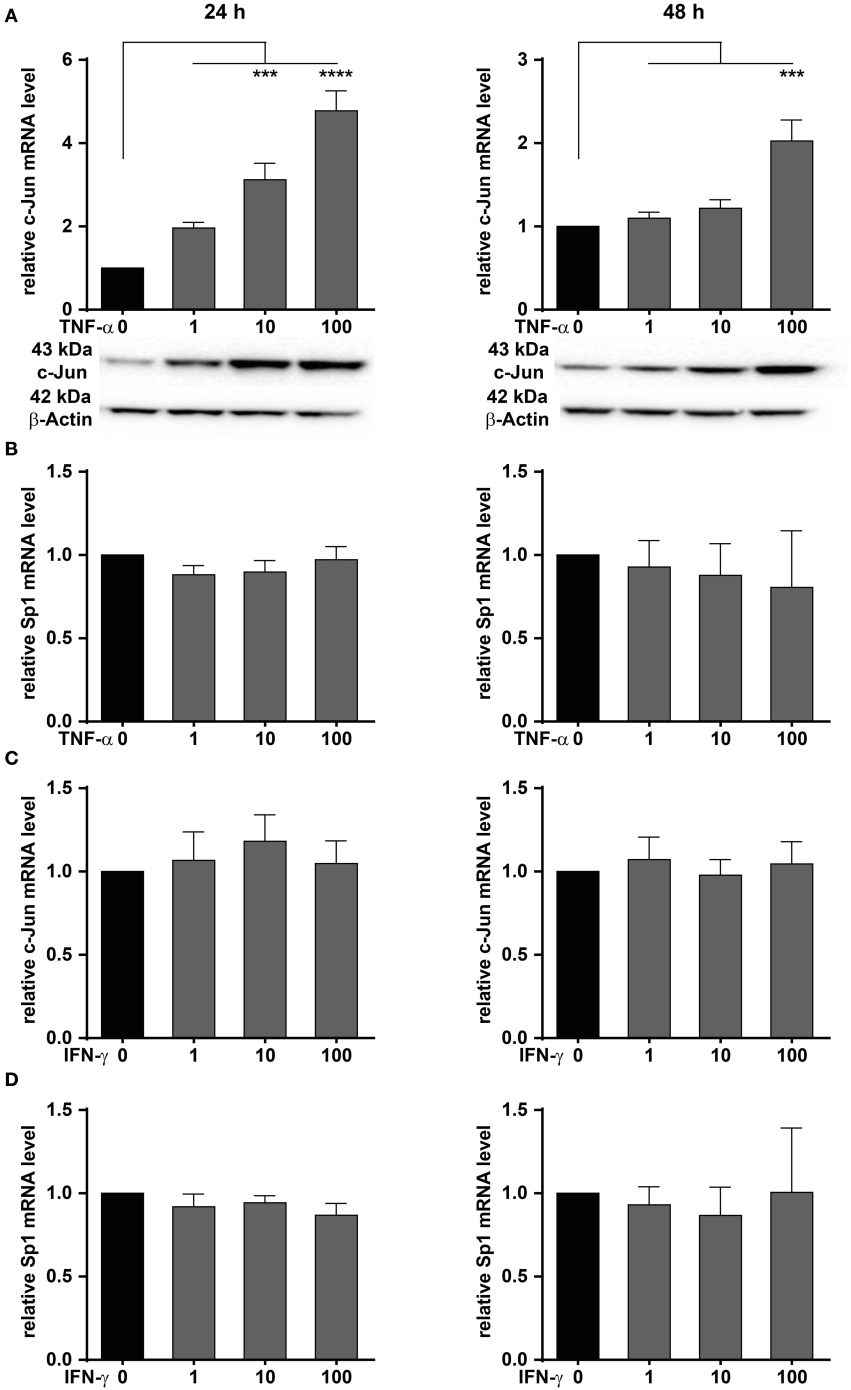
TNF-α increases c-Jun mRNA and protein expression. HCT116 CRC cells were treated with increasing concentrations of TNF-α **(A,B)** or IFN-γ **(C,D)** for 24 (left panels) and 48 h (right panels). Cells without TNF-α treatment served as controls. The mRNA and protein expression levels of c-Jun and Sp1 were measured by qRT-PCR. Western blot was used to confirm the upregulation at the mRNA level. TNF-α treatment induces c-Jun expression at the mRNA and protein level. Results are representative of at least three independent experiments. The data are presented as mean ± SEM with the statistical significance levels: ***p ≤ 0.001 and ****p ≤ 0.0001.

We have shown that MACC1 expression is regulated by the transcription factors AP-1 and SP1 (35). The transcription factor AP-1 is composed of two subunits with c-Jun being one of them. Here, the role of TNF-α on c-Jun activity driving MACC1 expression was investigated in CRC cells. The CRC cell line HCT116 was treated with increasing concentrations of TNF-α for 24 and 48 h. TNF-α potently stimulates c-Jun expression in a concentration-dependent manner at both the mRNA and protein level at 24 h (Figure 4A). The induction of c-Jun expression declined within 48 h after TNF-α application.

Besides c-Jun, the transcription factor Sp1 has been identified to bind and regulate the promoter of MACC1 (35). Like c-Jun, Sp1 activity can be regulated by TNF-α. HCT116 cells were treated with increasing concentrations of TNF-α for 24 and 48 h (Figure 4). Sp1 mRNA levels were subsequently determined by qRT-PCR. The mRNA levels of Sp1 were unchanged following TNF-α stimulation (Figure 4B). This suggests that TNF-α stimulation results in an increase of c-Jun transcription. In turn, elevated c-Jun protein levels led to increased MACC1 expression.

Since TNF-α treatment induced c-Jun, the role of the pro-inflammatory cytokine IFN-γ on the induction of c-Jun was also explored. HCT116 cells were treated with increasing concentrations of IFN-γ, and harvested after 24 or 48 h for analysis of c-Jun mRNA expression. No induction of c-Jun mRNA was seen in HCT116 cells (Figure 4C) following IFN-γ stimulation for 24 or 48 h. Similarly, the role of IFN-γ on the induction of Sp1 was also explored. Cells were treated with increasing concentrations of IFN-γ for 24 and 48 h (Figure 4D). Sp1 mRNA expression levels were subsequently analyzed by qRT-PCR. Similarly to TNF-α treatment, no induction of Sp1 mRNA expression was detected at any treatment concentration or time point. This indicates that IFN-γ has no effect on Sp1 expression. In conclusion, we demonstrated that TNF-α induces the expression of c-Jun, thereby impacting the control of MACC1 expression. Since TNF-α showed a stronger and more sustained effect on MACC1 gene expression and cell migration, this cytokine was further analyzed in more detail.

### TNF-α Regulates MACC1 Promoter Activity Through c-Jun/AP-1 Interacting With a Functional AP-1 Transcription Factor Binding Site

**Figure 5:**
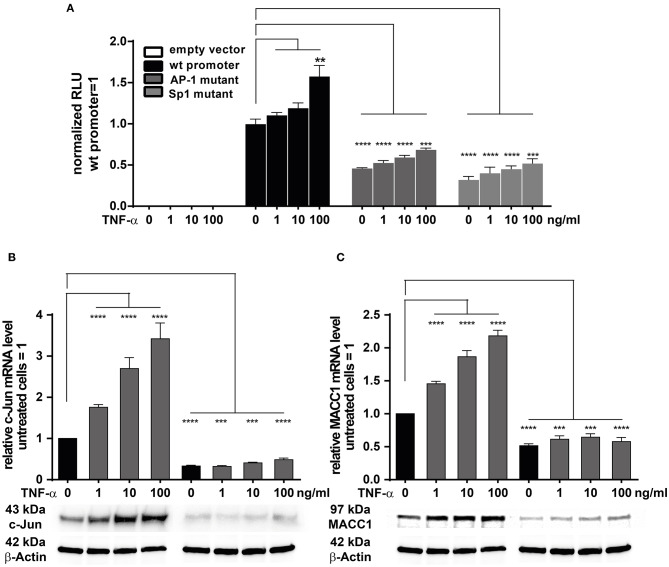
TNF-α induces MACC1 via c-Jun/AP1. **(A)** HCT116 cells were transiently transfected with the MACC1 promoter reporter plasmids with either mutated AP-1 or Sp1 transcription factor binding sites along with a renilla luciferase control plasmid for 24 h. Cells were then treated with increasing concentrations of TNF-α. After 24 h of TNF-α treatment luciferase activity was measured and normalized to renilla luciferase activity. **(B,C)** HCT116 cells were transfected with the target-specific predesigned c-Jun siRNA or scrambled control siRNA for 24 h. Cells were then stimulated with increasing concentrations of TNF-α for another 24 h. Cells without TNF-α treatment served as controls. Cells were analyzed to assess the c-Jun and MACC1 mRNA and protein expression levels using qRT-PCR and Western blotting, respectively. The data is presented as mean ± SEM with the statistical significance levels: **p ≤ 0.01, ***p ≤ 0.001, and ****p ≤ 0.0001.

We previously have cloned and described the MACC1 core promoter. We reported that MACC1 gene transcription relies on AP-1 and Sp1 protein activity and their respective promoter binding sites (35). As TNF-α induces c-Jun expression, a subunit of AP-1, we tested if this transcription factor has a direct role in MACC1 gene regulation after TNF-α stimulation. In parallel, Sp1 was also tested. We mutated the AP-1 and Sp1 transcription factor binding sites within the MACC1 promoter by site directed mutagenesis (35). HCT116 cells were transiently transfected with these AP-1 and Sp1 mutant promoter plasmids together with a renilla luciferase control plasmid for 24 h. Following TNF-α treatment for another 24 h, the luciferase activity as read out for the MACC1 promoter activity was analyzed using the Dual Luciferase reporter gene assay. Both the mutated AP-1 and Sp1 sites markedly reduced MACC1 promoter activity, accounting for the crucial role of the two binding sites for MACC1 promoter function (Figure 5A). TNF-α was able to induce the activity of the MACC1 promoter but failed to show this increase if the AP-1 or Sp1 binding site was mutated (Figure 5A). Since TNF-α was able to increase c-Jun but not Sp1 gene expression we tested, if siRNA mediated knock down of c-Jun impairs MACC1 gene expression and regulation by TNF-α (Figures 5B,C). Successful siRNA mediated c-Jun down regulation (Figure 5B) markedly reduced MACC1 gene expression (Figure 5C). Under these conditions, TNF-α treatment failed to increase c-Jun expression and subsequently MACC1 was not induced (Figures 5B,C). In summary, the AP-1 and Sp1 binding sites are indispensable elements for the transcriptional activation of the MACC1 gene. In the context of TNF-α stimulation, the induction of MACC1 relies on the functional AP-1 transcription factor binding site.

### Signaling Through TNFR1 and NF-κB Activates c-Jun for MACC1 Induction

**Figure 6:**
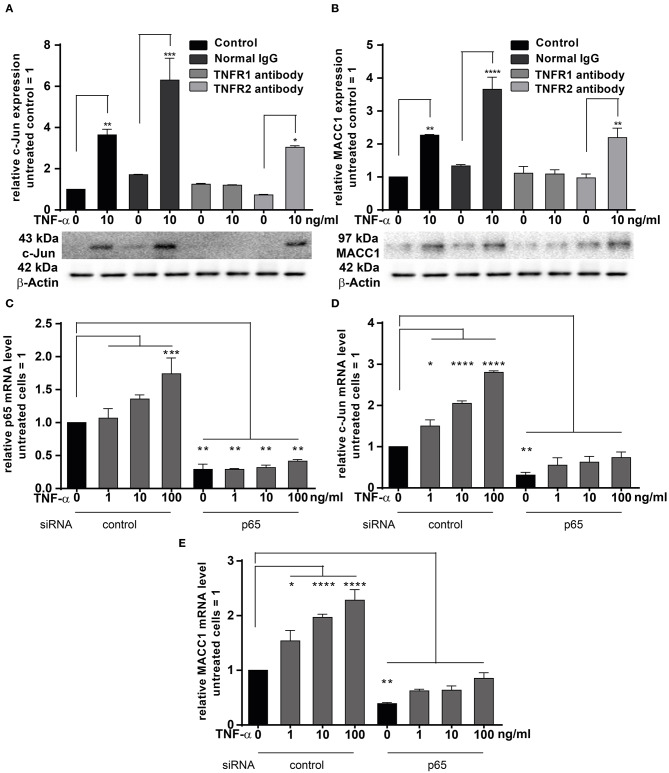
TNFRI is the major receptor responsible for TNF-α mediated MACC1 induction. **(A,B)** One hour prior treatment with 10 ng/ml TNF-α HCT1116 cells were pre-incubated with specific blocking antibodies targeting TNFR1 or TNFR2 for 24 h. Cells were harvested and analyzed to assess the c-Jun **(A)** and MACC1 **(B)** mRNA and protein expression levels using qRT-PCR and Western blot, respectively. Isotype IgG antibodies not targeting the TNF receptors served as negative controls. **(C–E)** HCT116 cells were transfected with p65 siRNA or scrambled control for 24 h. Cells were then treated with increasing concentrations of TNF-α for another 24 h. Total RNA was extracted, reverse transcribed and the mRNA levels of p65 **(C)**, c-Jun **(D)**, and MACC1 **(E)** were quantified using qRT-PCR. The data are presented as mean ± SEM with the statistical significance levels: *p ≤ 0.05, **p ≤ 0.01, ***p ≤ 0.001 and ****p ≤ 0.0001.

TNF-α exerts its effects through binding to two membrane receptors, TNFR1 or TNFR2 (36–38). These receptors show different expression patterns: TNFR1 is extensively expressed in many cell types; but TNFR2 shows a limited expression range and is selectively found in immune and endothelial cells (38). Since TNF-α triggers MACC1 expression, we were interested in identifying the responsible receptor mediating this effect in cancer cells. To identify the responsible receptor in our model system, HCT116 cells were pre-incubated with specific blocking antibodies for either TNFR1 or TNFR2 for 1 h. Afterwards, the cells were stimulated with 10 ng/ml TNF-α. Following 24 h of TNF-α treatment, cells were harvested and analyzed for c-Jun and MACC1 expression both at the mRNA and protein levels. TNF-α stimulation upregulated both c-Jun and MACC1 expression in the control group. However, the upregulation of c-Jun (Figure 6A) disappeared at both the mRNA and protein level upon pretreatment with a TNFR1-specific blocking antibody. Contrary, TNF-α treatment successfully upregulated c-Jun expression, despite pretreatment with TNFR2-specific blocking antibodies. This shows, that TNFR2 has only a minor role in regulating MACC1 expression after TNF-α stimulation. In accordance with the c-Jun expression pattern, the mRNA and protein expression levels of MACC1 (Figure 6B) showed no increase after TNF-α treatment upon pretreatment with a TNFR1-specific blocking antibody. As for c-Jun, MACC1 expression was still up-regulated upon pretreatment with a TNFR2-specific blocking antibody.

The pro-inflammatory NF-κB signaling is activated by at least three pathways (39). One of these pathways is the so-called “canonical” pathway triggered by TNF-α, which results in the activation of p65 that regulates the inflammatory responses (40). HCT116 cells were transfected with siRNA targeting p65 for 24 h. The cells were treated with increasing concentrations of TNF-α for another 24 h. Unstimulated cells served as controls. The mRNA expression levels of p65 (Figure 6C) were increased in a concentration-dependent manner by TNF-α treatment. Successful knock down of p65 abolished the induction of p65 by TNF-α stimulation. Next, the mRNA expression levels of c-Jun and MACC1 were examined. Again, both proteins were up-regulated by TNF-α treatment in a dose-dependent manner. Knock down of p65 abated basal mRNA expression levels of c-Jun (Figure 6D) and MACC1 (Figure 6E). The cells with p65 knock down showed only a marginal dose-dependent response to TNF-α treatment.

In conclusion, TNF-α executed c-Jun and MACC1 induction through TNFR1, but not TNFR2. Blocking TNFR1, but not TNFR2, inhibited both c-Jun and MACC1 induction by TNF-α at the mRNA and protein level. Additionally, c-Jun and MACC1 mRNA expression were inhibited by knock down of p65, indicating that the canonical NF-κB pathway is directly involved in the induction of c-Jun that regulates the MACC1 gene.

### Adalimumab Can Reverse the TNF-α Induced MACC1 Expression

**Figure 7:**
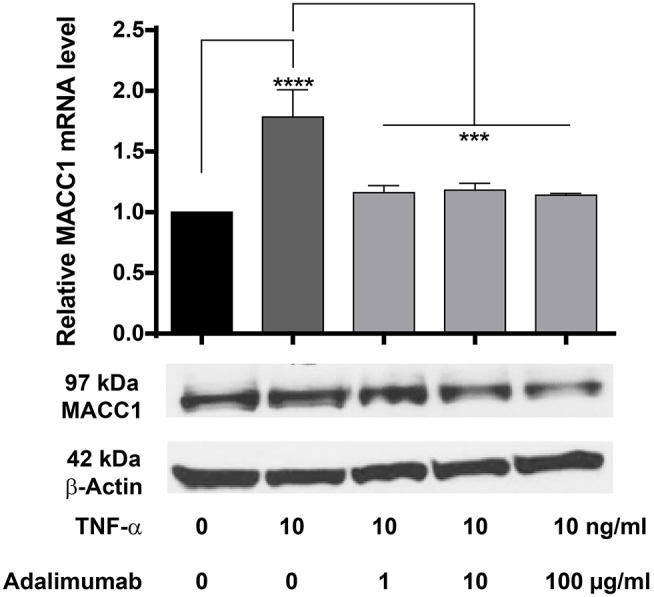
Effect of TNF-α and the TNF-α neutralizing antibody adalimumab treatment on MACC1 mRNA and protein expression. HCT116 cells were treated with 10 ng/ml TNF-α and increasing concentrations of adalimumab (1, 10, 100 μg/ml) for 24 h before RNA isolation and qRT-PCR. TNF-α increased MACC1 gene expression at the mRNA and protein level that was abolished by blocking TNF-α with adalimumab. The data are presented as mean ± SEM with the statistical significance levels: ***p ≤ 0.001 and **** *p* ≤ 0.0001.

Adalimumab is a clinically approved TNF-α neutralizing monoclonal antibody applied widely in the treatment of chronic inflammatory diseases including Crohn's disease and ulcerative colitis. We therefore tested, if adalimumab can inhibit the TNF-α induced MACC1 induction. HCT116 cells were co-administered with 10 ng/ml TNF-α and increasing concentrations of adalimumab before MACC1 mRNA and protein expression was determined via qRT-PCR and Western blot, respectively. Compared to control cells, adalimumab treatment resulted in a significant decrease in MACC1 gene expression at all adalimumab concentrations tested (Figure 7). In addition we tested if adalimumab can revert the TNF-α effect in the wound healing (scratch) assay. Cellular motility is increased if cells are stimulated with TNF-α (Supplementary Figure 2A). If the cells are treated with adalimumab in parallel this effect is reverted to control levels (Supplementary Figure 2B).

These data confirm our previous findings that TNF-α increases MACC1 expression. More importantly, it demonstrates that adalimumab effectively inhibits TNF-α action and reduces its effect on MACC1 expression.

## Discussion

The close connection of inflammation and cancer is long known (41), but how inflammatory processes drive cancer development and progression is not thoroughly described. Here we report, that MACC1, a prognostic and predictive marker for numerous solid cancer entities, is increased in inflamed tissues. We analyzed in detail, how major pro-inflammatory cytokines mediate this elevated MACC1 gene expression leading to increased cellular motility. Most importantly, we show that the clinically approved TNF-α blocking antibody adalimumab can prevent the increase in MACC1 gene expression, offering a potential treatment option for patients.

The connection of inflammation and cancer, particularly CRC, involving pro-inflammatory cytokines was shown by numerous studies (42–44). Although the link of inflammation and cancer metastasis is already described, the cell specific and inflammation induced molecular mechanisms enabling cancer cells to metastasize are not thoroughly described (45–47). Expression of the MACC1 gene, particularly in CRC, can result in tumor invasion and metastasis. It is not known, why MACC1 expression increases during tumor development. It was demonstrated that MACC1 expression can be induced by IL-4 and lipopolysaccharide (LPS) in bone marrow-derived macrophages, suggesting that MACC1 might be involved in inflammatory processes (48). Therefore, an examination of the MACC1 gene regulation, particularly during inflammation, can help to clarify the relationship between inflammation, carcinogenesis and metastasis in CRC.

We have shown that MACC1 expression is increased in inflamed tissue of ulcerative colitis and Crohn's disease patients. It is well accepted that TNF-α and IFN-γ are major players in the pathogenesis of these chronic diseases (49). Therefore, we hypothesized that these pro-inflammatory cytokines regulate MACC1 gene expression in CRC cells. We demonstrated here, that particularly TNF-α regulates MACC1 at both the transcriptional and translational level in a time- and dose-dependent manner. Thus, the chronic inflammatory microenvironment sustained by TNF-α might be an important condition of CRC progression. Inflammation regulates many aspects of cancer progression like proliferation, angiogenesis, invasion, and metastasis (50). For different tumor entities, not only time but cytokine concentration decides about molecular outcome (51, 52). We found that TNF-α concentrations affect levels of MACC1 mRNA and protein expression in a dose-dependent manner.

Increased MACC1 expression leads to cellular motility *in vitro* and metastasis *in vivo* (29). TNF-α was demonstrated as inducer of cell migration in cancer cells (53). TNF-α can contribute to migration of CRC cells through the epithelial-mesenchymal transition (EMT) (54). This process is further promoted by the combined activity of pro-inflammatory cytokines and MACC1. We found that low concentrations of TNF-α augment MACC1-induced cell migration, whereas high doses of TNF-α hinders cell migration in CRC cells overexpressing MACC1. In this setting cell death overrules the stimulating effects of TNF-α (55). Silencing of MACC1 mRNA abrogates the effects of TNF-α on cell migration and precludes cell responsiveness to TNF-α treatment. Hence, TNF-α increases cell migration by acting besides other factors, through the MACC1 gene, thereby augmenting the migratory potential of MACC1 in CRC.

The transcription factor c-Jun is stimulated by TNF-α through c-Jun N-terminal kinase (JNK) (56). This classical signaling pathway is known to be involved in inflammation and cancer (57, 58). We analyzed c-Jun mRNA and protein levels in response to TNF-α treatment and found that TNF-α induced transcription and translation of c-Jun in a dose-dependent manner in CRC cells. Hence, TNF-α can facilitate a variety of pathophysiological activities directly or indirectly by regulating c-Jun expression. This pathway is not only relevant for CRC, but for other tumor entities as well, like hepatocellular carcinoma, pancreatic cancer or nasopharyngeal carcinoma (59–61).

The c-Jun protein increased by TNF-α is part of the transcription factor AP-1 that was identified to drive MACC1 gene expression. The core promoter of MACC1 was identified between the nucleotides −992 to −18 relative to the transcriptional start site. This region drives transcription of the MACC1 gene with most of the regulatory features (35). The minimal essential core promoter region of MACC1 lies within nucleotides −426 to −18. It encompasses all sequences needed for MACC1 transcription, including initiation of transcription and basal activation of the MACC1 gene. The core promoter region contains functional binding sites for transcription factors, including AP-1, Sp1, and C/EBPs, which were shown to regulate MACC1 expression (35).

TNF-α mediates a variety of cell-signaling processes involved in the immune response and carcinogenesis, primarily via its interaction with TNFR1 and/or TNFR2 (62, 63). TNFR1 is a central regulator of signal transduction pathways whereas TNFR2 is expressed on a very narrow subset of immune cells (64–66). Based on our previous study on the effects of TNF-α on c-Jun/MACC1 signaling, we exposed CRC cells to blocking antibodies for TNFR1 or TNFR2, respectively. Blocking of TNFR1 did not change the basal MACC1 expression level but caused a loss of responsiveness of c-Jun and MACC1 mRNA and protein expression to TNF-α stimulation. In contrast, exposure to anti-TNFR2 antibodies did not preclude the stimulation of c-Jun and MACC1 by TNF-α. These results show that TNF-α induces c-Jun and MACC1 via TNFR1 signaling, but not TNFR2. Thus, these findings confirm a signaling axis comprising TNFR1 and c-Jun, leading to MACC1 expression that eventually mediates tumor progression and metastasis.

TNF-α induces NF-κB to activate signal transductions processes. NF-κB is a multifunctional transcription factor with essential roles in a variety of biological activities and cellular responses. NF-κB subunits form various homo- and heterodimers. In the canonical pathway, NF-κB is activated by pro-inflammatory cytokines, such as TNF-α (67).

Consistent with previous studies, we determined that TNF-α activates c-Jun to regulate the induction of MACC1 in CRC cells. We explored the effects of NF-κB signaling on c-Jun and MACC1 by knocking down p65. Our results showed that TNF-α increases the levels of p65 mRNA expression in a dose-dependent manner. In the context of p65 knockdown, the basal levels of c-Jun and MACC1 mRNA were lower and the TNF-α responsiveness was mainly lost. Therefore, the canonical NFκB pathway induces via p65—a subunit of NF-κB—directly or indirectly the transcription of c-Jun and controls the induction of MACC1 in CRC cells. Our findings indicate a notable signaling network involved in cancer development.

TNF-α activates NF-κB signaling, thereby contributing to inflammation, cell survival, proliferation, angiogenesis, tumor promotion, and metastasis (68, 69). The transcription factor NF-κB links inflammatory signaling and cancer. It is involved in nearly every stage of cancer development, including invasion and metastasis. NF-κB promotes tumor metastasis by regulating epithelial mesenchymal-transition (EMT) in CRC (70, 71). Furthermore, TNF-α, secreted by pro-inflammatory macrophages, enhances the metastatic potential of ovarian tumor cells via activation of the NF-κB signaling pathway (72).

With TNF-α/TNFR1, confidently established as an inducer of MACC1, we investigated whether a clinically approved TNF-α blocking antibody would prevent the induction of MACC1. The human TNF-α blocking monoclonal antibody adalimumab was used. Adalimumab is used in the treatment of a number of chronic inflammatory diseases, including rheumatoid arthritis, colitis ulcerosa or Crohn's disease. Adalimumab has been shown to induce apoptosis of human macrophages while down regulating levels of soluble TNF-α as well as other pro-inflammatory cytokines (73–75). Here we show that adalimumab reduces TNF-α induced MACC1 over-expression. This indicates that a TNF-α specific antibody could be effective for treatment of MACC1 driven tumors. Interfering with MACC1 expression via TNF-α could prove to be a valuable additional therapeutic strategy against CRC metastasis.

Taken together, our findings support the hypothesis that the transcription factors c-Jun and NF-κB can be considered as a potential molecular target in CRC therapy for MACC1 driven tumors. Control of inflammation offers an effective approach for repressing or maybe even preventing tumor metastasis.

## Data Availability Statement

All datasets generated for this study are included in the article/Supplementary Material.

## Ethics Statement

The studies involving human participants were reviewed and approved by institutional review board of the Charité–Universitätsmedizin Berlin. The patients/participants provided their written informed consent to participate in this study.

## Author's Note

Parts of this study were used in a dissertation thesis conducted at the Experimental and Clinical Research Center, Charité—Universitätsmedizin Berlin, and Max-Delbrück-Center for Molecular Medicine, Berlin-Buch (76).

## Author Contributions

DK, RG, BS, and US: study conception and design. CZ, DK, and IC-L: conducted experiments. DK, CZ, IC-L, and US: drafting the manuscript. All authors: analysis and interpretation of data and critical revision.

## Conflict of Interest

BS has served as Consultant for Abbvie, Boehringer, Celgene, Falk, Janssen, Lilly, Pfizer, Prometheus, Takeda and received speaker's fees from Abbvie, CED Service GmbH, Falk, Ferring, Janssen, Novartis, Takeda (BS served as representative of the Charité). The remaining authors declare that the research was conducted in the absence of any commercial or financial relationships that could be construed as a potential conflict of interest.

## Supplementary Material

Supplementary Figure 1 Effects of TNF-α on the MACC1 gene expression in different cell lines. Three different established cells and primary cell cultures were treated with increasing concentrations of TNF-α (1, 10, 100 ng/ml). Cells without cytokine treatment served as controls. MACC1 mRNA expression levels were determined by qRT-PCR. The pro-inflammatory cytokine TNF-α can upregulate MACC1 gene expression in a dose-dependent manner. The experiments were performed as three biologically independent experiments. The data are presented as mean ± SEM with the statistical significance levels: ^*^*p* ≤ 0.05; ^**^*p* ≤ 0.01.

Supplementary Figure 2 TNF-α increases cellular motility in the wound healing (scratch) assay that is reverted by adalimumab. HCT116 cells were seeded at a density of 1.1 × 10^6^ cells per ml in 96-well image lock plates. The cells were allowed to adhere for 6 h forming a confluent monolayer. Wounds (scratches) were applied using the wound maker tool. Directly after wounding the cells were treated with increasing amounts of TNF-α (1, 10, and 100 ng/ml) alone or in combination with 100 μg/ml adalimumab. The cells were monitored label-free every second hour in the IncuCyte live cell imaging system. TNF-α increased wound closure in a dose-dependent manner over time **(A)**. This phenotype could be reverted by adalimumab **(B)**.
