# Supplementary figures and images for: Pro-inflammatory TNF-α and IFN-γ Promote Tumor Growth and Metastasis via Induction of MACC1
Source: Front Immunol. 2020 May 27;11:980. doi: 10.3389/fimmu.2020.00980 (PMC7326113; doi:10.3389/fimmu.2020.00980)

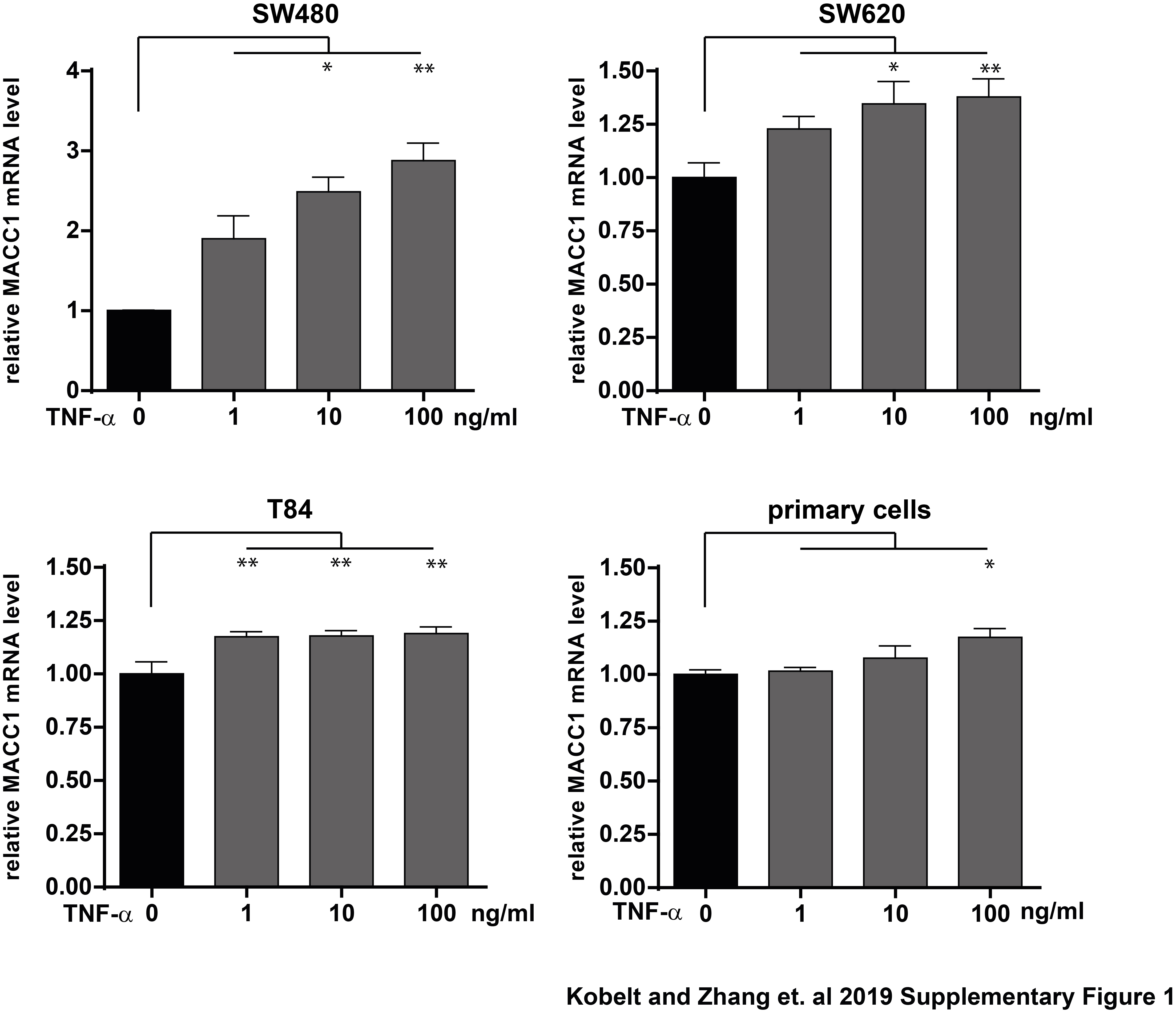

Supplement: Supplementary Figure 1 — Effects of TNF-α on the MACC1 gene expression in different cell lines. Three different established cells and primary cell cultures were treated with increasing concentrations of TNF-α (1, 10, 100 ng/ml). Cells without cytokine treatment served as controls. MACC1 mRNA expression levels were determined by qRT-PCR. The pro-inflammatory cytokine TNF-α can upregulate MACC1 gene expression in a dose-dependent manner. The experiments were performed as three biologically independent experiments. The data are presented as mean ± SEM with the statistical significance levels: *p ≤ 0.05; **p ≤ 0.01. [file Image_1.JPEG]

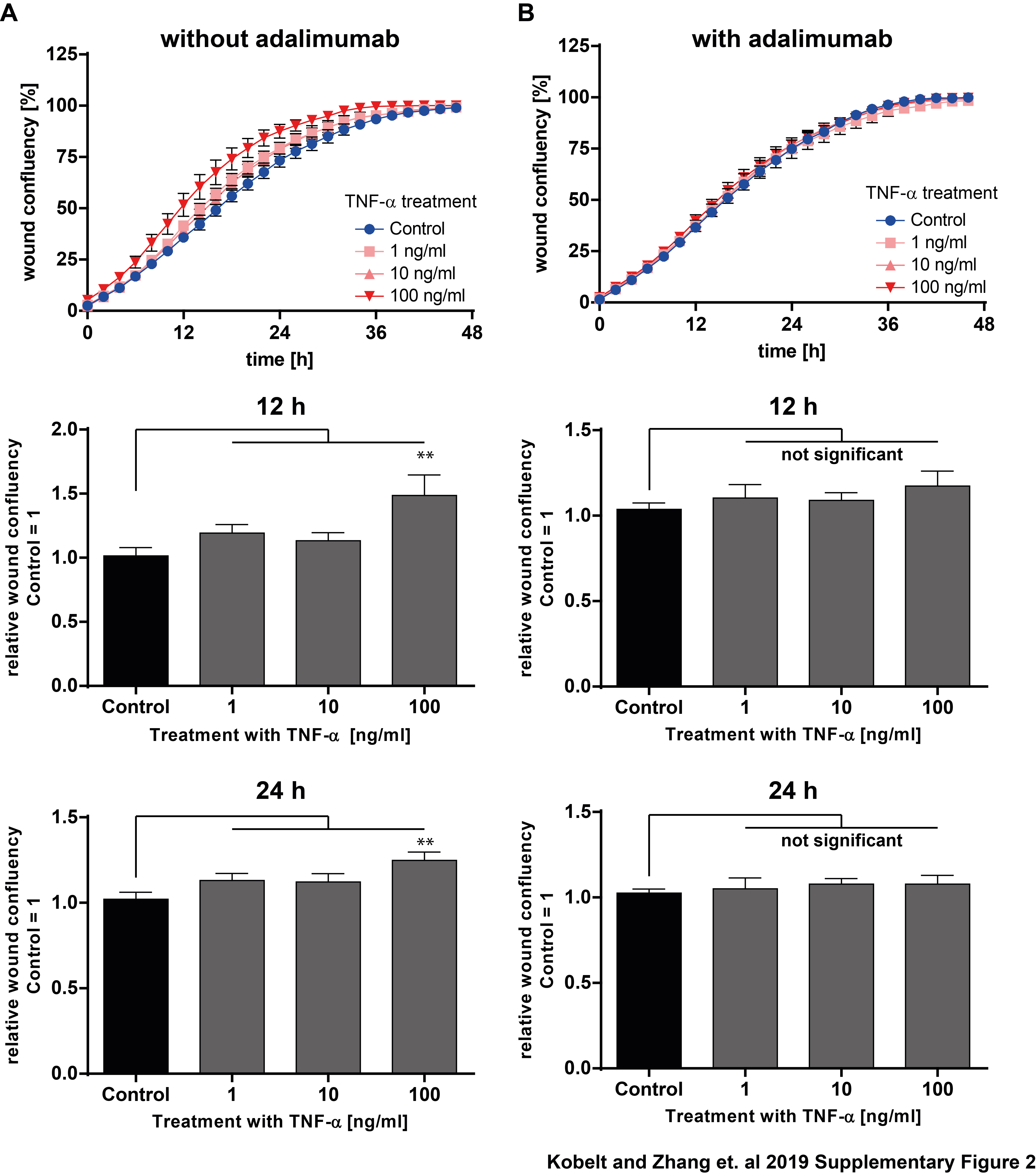

Supplement: Supplementary Figure 2 — TNF-α increases cellular motility in the wound healing (scratch) assay that is reverted by adalimumab. HCT116 cells were seeded at a density of 1.1 × 10^6^ cells per ml in 96-well image lock plates. The cells were allowed to adhere for 6 h forming a confluent monolayer. Wounds (scratches) were applied using the wound maker tool. Directly after wounding the cells were treated with increasing amounts of TNF-α (1, 10, and 100 ng/ml) alone or in combination with 100 μg/ml adalimumab. The cells were monitored label-free every second hour in the IncuCyte live cell imaging system. TNF-α increased wound closure in a dose-dependent manner over time (A). This phenotype could be reverted by adalimumab (B). [file Image_2.JPEG]
